# Delivering water, sanitation and hygiene interventions to women and children in conflict settings: a systematic review

**DOI:** 10.1136/bmjgh-2019-002064

**Published:** 2020-07-08

**Authors:** Daina Als, Sarah Meteke, Marianne Stefopulos, Michelle F Gaffey, Mahdis Kamali, Mariella Munyuzangabo, Shailja Shah, Reena P Jain, Amruta Radhakrishnan, Fahad J Siddiqui, Anushka Ataullahjan, Zulfiqar A Bhutta

**Affiliations:** 1 Centre for Global Child Health, Hospital for Sick Children, Toronto, Ontario, Canada; 2 Health Services and Systems Research, Duke-NUS Graduate Medical School, Singapore; 3 Center of Excellence in Women and Child Health, Aga Khan University, Karachi, Pakistan

**Keywords:** hygiene, maternal health, public health, treatment, systematic review

## Abstract

**Background:**

Access to safe water and sanitation facilities and the adoption of effective hygiene practices are fundamental to reducing maternal and child morbidity and mortality globally. In armed conflict settings, inadequate water, sanitation and hygiene (WASH) infrastructure poses major health risks for women and children. This review aimed to synthesise the existing information on WASH interventions being delivered to women and children in conflict settings in low-income and middle-income countries (LMICs) and to identify the personnel, sites and platforms being used to deliver such interventions.

**Methods:**

We conducted a systematic search for publications indexed in four databases, and grey literature was searched through the websites of humanitarian agencies and organisations. Eligible publications reported WASH interventions delivered to conflict-affected women or children. We extracted and synthesised information on intervention delivery characteristics, as well as barriers and facilitators.

**Results:**

We identified 58 eligible publications reporting on the delivery of WASH interventions, mostly in Sub-Saharan Africa. Non-Governmental Organization (NGO)/United Nations (UN) agency staff were reported to be involved in delivering interventions in 62% of publications, with the most commonly reported delivery site being community spaces (50%). Only one publication reported quantitative data on intervention effectiveness among women or children.

**Discussion:**

This review revealed gaps in the current evidence on WASH intervention delivery in conflict settings. Little information is available on the delivery of water treatment or environmental hygiene interventions, or about the sites and personnel used to deliver WASH interventions. Limited quantitative data on WASH intervention coverage or effectiveness with respect to women or children are important gaps, as multiple factors can affect how WASH services are accessed differently by women and men, and the hygiene needs of adolescent girls and boys differ; these factors must be taken into account when delivering interventions in conflict settings.

**PROSPERO registration number:**

CRD42019125221

**Key questions**

**What is already known?**

- Populations affected by conflict are at increased risk of poor health outcomes as a result of inadequate access to water, sanitation and hygiene (WASH) facilities and resources.
- Increased population displacement caused by conflict often leads to overcrowding in camps, creating optimal conditions for the transmission of communicable diseases.
- Provision of clean water and improved sanitation facilities have been successful methods of improving maternal and newborn child health in conflict settings.

**What are the new findings?**

- There is a lack of high-quality information and data on the delivery and effectiveness of WASH interventions for women and children in conflict settings.
- Many WASH interventions are reported to be delivered in community settings, but there may still be missed opportunities for delivering WASH interventions particularly to school-aged children, including the use of teachers in the delivery of hygiene promotion interventions and the distribution of soap/hygiene kits in schools or other educational settings in or outside of camps.
- Poor coordination between relief agencies is a key barrier to delivering WASH interventions in conflict settings.

**What do the new findings imply?**

- Better documentation and more research are needed on the delivery and effectiveness of WASH interventions for conflict-affected children, adolescents and pregnant and lactating women.

## Introduction

Water, sanitation and hygiene (WASH) are fundamental determinants of an individual’s overall health,^1^ with access to safe water and sanitation facilities and adoption of effective hygiene practices playing important roles in the prevention of morbidity and mortality globally, particularly among children.^2^ Various organisations have been working to increase access to WASH services and reduce unsafe water-related mortality for decades. Despite these efforts, in 2015, the WHO and United Nations Children's Fund (UNICEF) Joint Monitoring Programme estimated that globally, 844 million people were without basic drinking water services, 2.3 billion lived without basic sanitation facilities and just under 900 million people were practicing open defecation.^3^

War and conflict are responsible for the forcible displacement of more than 17 million children as of 2017,^4^ and nearly 50% of the world’s refugee population in 2018 was comprised of women and young girls.^5^ In conflict-affected populations, alongside mass displacement, people are also at risk of exposure to collapsing infrastructure, food insecurity, unsafe water and insufficient water supply as well as inadequate sanitation facilities. Among refugees and internally displaced persons (IDPs), overcrowding in camps and inadequate WASH infrastructure increase the risks of diarrhoea, cholera and infection from parasites such as soil-transmitted helminths, further perpetuating the risk of fecal–oral disease transmission.^6 7^ Additionally, women and children face an increased risk of sexual and physical violence^8^ as well as work/school absenteeism^9^ as a result of inadequate or complete lack of sanitation facilities and poor menstrual hygiene management.

An estimated 16% of the world’s children were living in conflict-affected areas in 2016.^10^ Recent analyses of data from 35 African countries found that conflict within 50 km of a child’s dwelling was associated with a 7.7% increase in the risk of dying in the first year of life,^11^ with conflict also posing increased mortality risk for women and mothers in these unstable environments, especially indirectly through the breakdown of health and other infrastructure.^12^ Among children under 5, the number of deaths indirectly attributable to conflict were three to five times higher than directly attributable deaths^11^; damaged or deteriorated WASH infrastructure will have been a driver of least some of this indirect conflict mortality.

This review is one of a series of reviews examining health and nutrition intervention delivery to conflict-affected women and children in low- and middle-income countries (LMICs). The aim of the present review was to synthesise information from the indexed and grey literature on the delivery of WASH interventions to women and children in conflict settings. The primary objective was to synthesise information on how WASH interventions have been delivered to conflict-affected women and children, with a focus on personnel, platforms and sites, with a secondary objective of synthesising the available evidence on achieved intervention coverage and effectiveness for those women and children. A third objective was to synthesise reported information on factors affecting intervention delivery, either positively or negatively.

## Methods

The protocol for this review is registered with PROSPERO, and its reporting adheres to PRISMA statement (online supplementary appendix A).

10.1136/bmjgh-2019-002064.supp1 Supplementary data

### Indexed literature search

A systematic search of literature published from 1 January 1990 to 31 March 2018 was conducted in MEDLINE, Embase, CINAHL and PsycINFO using OVID and EBSCO interfaces and sets of search terms related to three concepts: (1) conflict, (2) women and children and (3) water, sanitation and hygiene. Conflict-related terms included war, crisis, refugees and IDP. Population-related words included women, children, pregnant, adolescents and newborn. WASH-related terms included drinking water, hygiene, hand washing, human excreta disposal and latrines. The complete MEDLINE search syntax is presented in online supplementary appendix B. The reference lists of relevant systematic reviews conducted in the last decade were also screened, including a 2015 review by Ramesh *et al* 6 on WASH interventions and health outcomes in humanitarian crises, which informed the development of the search syntax for the present review.

For grey literature, we searched the websites of 14 major humanitarian agencies and organisations which are actively involved in researching or responding to conflict situations for reports on the delivery of health interventions to our populations of interest: Action Contre la Faim, Care International, Emergency Nutrition Network, Oxfam International, International Committee of the Red Cross, International Rescue Committee, Médecins Sans Frontières, Save the Children, Solidarités International, United Nations Population Fund (UNFPA), United Nations High Commissioner for Refugees (UNHCR), UNICEF, Women’s Refugee Commission and World Vision. We used broad terms for conflict and health interventions tailored to the search functionality of each website. Because of the large volume of grey literature available, we further restricted eligible grey literature publications to those published since 1 January 2013, in order to be able to screen and assess them feasibly. Exact publication dates are rarely reported for the grey literature and so we were unable to truncate our grey literature search to align exactly with the indexed literature search period; we therefore screened all grey literature published up to 30 November 2018, the date of our grey literature search.

### Eligibility criteria

Eligible publications were limited to those reporting on populations affected by conflict in LMICs, as classified by the World Bank in 2017,^13^ and describing a WASH intervention being delivered during or within 5 years of cessation of a conflict. Where needed, we consulted online encyclopaedic sources as well as the UN Office for the Coordination of Humanitarian Affairs (OCHA)^14^ website for information on the duration of a specific conflict, to assess whether the time period of intervention delivery reported in a candidate publication was eligible. For the purpose of this review, WASH interventions included those aiming to provide clean water (eg, establishing household connections, construction of hand pumps, water distribution points) or improve water quality (eg, source-based water treatment, chlorine-based water treatment, improving water storage, filtration, UV treatment), improve or provide sanitation facilities (eg, flush or pour toilets to piped sewerage system, pit latrines, ventilated improved latrine) or promote hygiene (eg, enforcing hand washing with soap at critical times, health promotion campaigns, hygiene education, mass media campaigns).^15^ An eligible intervention was required to target or include neonates, children, adolescents or women of reproductive age. General population interventions were therefore included as our target populations were among the beneficiaries. In order to identify the most informative resources from the large volume of grey literature available, the same eligibility criteria were applied, with the additional requirement of explicit reporting on the delivery site and personnel for each intervention.

Non-English publications, publications reporting on male populations exclusively, case reports of a single patient, studies on military personnel, refugee populations bound for a high-income country, or surgical techniques and pure economic or mathematical modelling studies were excluded from our review. Other exclusion criteria included systematic reviews, guidelines and studies where no specific health intervention was described (eg, prevalence studies).

### Data extraction and analysis

All retrieved indexed records were downloaded into EndNote X7 software^16^ and duplicates were removed. Unique records were then imported into Covidence software for screening. Titles and abstracts were reviewed in duplicate, and the full-text reports of potentially relevant publications were screened by a single reviewer who noted reasons for exclusion. Information and data from indexed and grey literature publications meeting the eligibility criteria were extracted in duplicate by two reviewers independently, using a customised form in Research Electronic Data Capture (REDCap)^17^ software hosted at The Hospital for Sick Children. We extracted information and data on setting and population characteristics, as well as key intervention delivery characteristics including delivery platform, personnel and site. These delivery characteristics were our main outcomes of interest. We extracted quantitative data on intervention coverage and effectiveness for women and children where available, as secondary outcomes. We also extracted information on reported delivery barriers and facilitators from those publications reporting on interventions targeting women or children specifically. The double-entered data were compared using REDCap software tools and any inconsistencies were resolved by discussion or by a third reviewer from among the coauthors, if needed.

We tabulated and plotted counts and proportions to summarise key characteristics of the literature including publication type, target settings, target populations including population displacement status, delivered interventions and delivery characteristics. We tabulated available quantitative data on intervention coverage and effectiveness relating to women, children or adolescents specifically; given the extremely limited quantitative data reported for these groups, we could not undertake meta-analysis. Information on barriers and facilitators was synthesised narratively, by grouping reported factors that had positively or negatively affected intervention delivery into common themes.

## Results

### Characteristics of included publications

**Figure 1:**
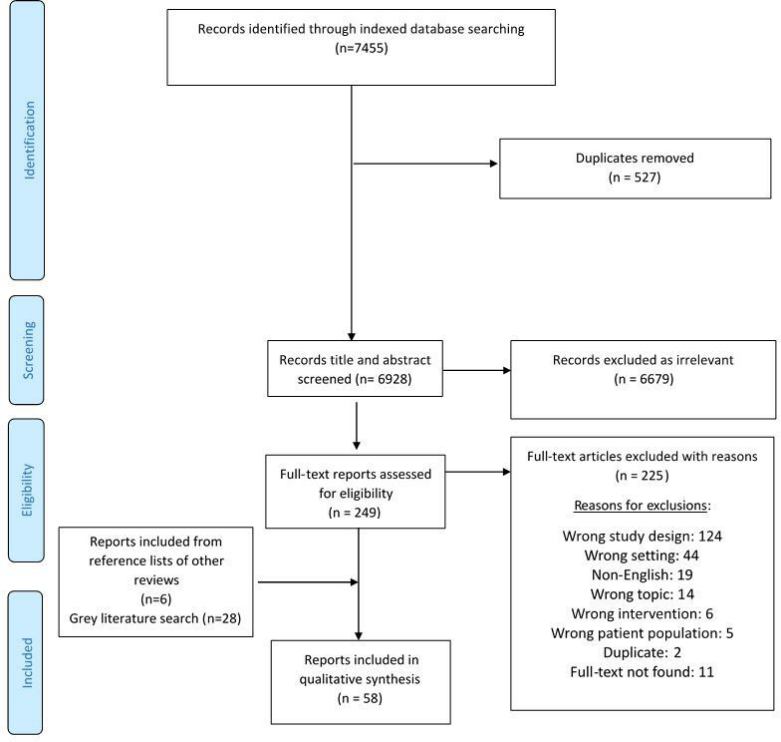
Flow diagram of included publications.

**Figure 2:**
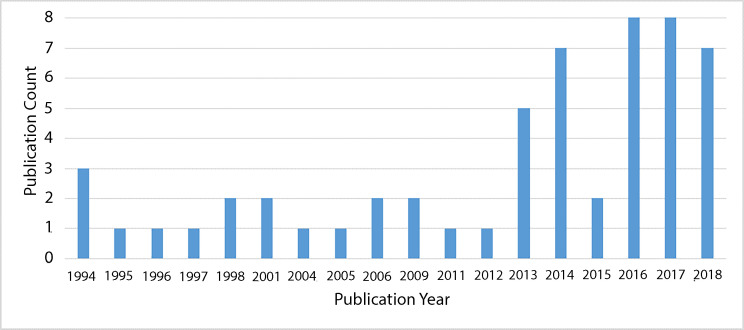
Distribution of included publications by publication year.

Our indexed database search returned 7455 records, and 30 of these publications were assessed as meeting our review eligibility criteria. The flow of literature screening and selection is presented in figure 1. An additional 28 eligible publications were identified from grey literature sources, for a total of 58 publications included in this review. More than half of the included literature was published from 2011 onward, with over a quarter published in 2017 and 2018 (figure 2). Publications that did not report on studies aiming to answer specific research questions were classified as non-research reports, including NGO reports of programme implementation. Most eligible publications were non-research reports (40/58, 69%), and observational research studies made up just under one-third of the included literature (table 1; full characteristics of included publications are presented in online supplementary appendices C and D).

**Table 1:** Summary characteristics of included publications (n=58)

Geographic Region^*^	**n**
East Asia and Pacific	10
Europe and Central Asia	0
Latin America and the Caribbean	0
Middle East and North Africa	13
South Asia	10
Sub-Saharan Africa	42
**Publication type**	**n**
Non-research report	39
Mixed methods	2
Observational study	14
Qualitative study	1
Quasi-experimental study	0
Randomised controlled trial	2
**Target population type** ^*^	**n**
All/General population	48
Women of reproductive age	6
Adolescents (10–19 years)	8
**Displacement status of beneficiary population** ^*^	**n**
Refugees	36
IDPs	28
Non-displaced	5
Returning refugees	5
Host	11
Unreported	3
**Setting of displaced populations** ^**†**^	**n**
Camp	27
Dispersed	5
Mixed	17
Unreported	9
**Delivery platform** ^*^	**n**
Existing Health System	14
Faith-based system	1
Informal governance	0
NGO/UN agencies	53
Military based	0
Research based	2
Mass Media	1

*Publications can be in more than one category.

†Only reflects publications that reported displaced status for populations (refugees, IDPs or returning refugees).

IDPs, internally displaced persons; NGO, Non-Governmental Organization; PLW, pregnant and lactating women; UN, United Nations.

**Figure 3:**
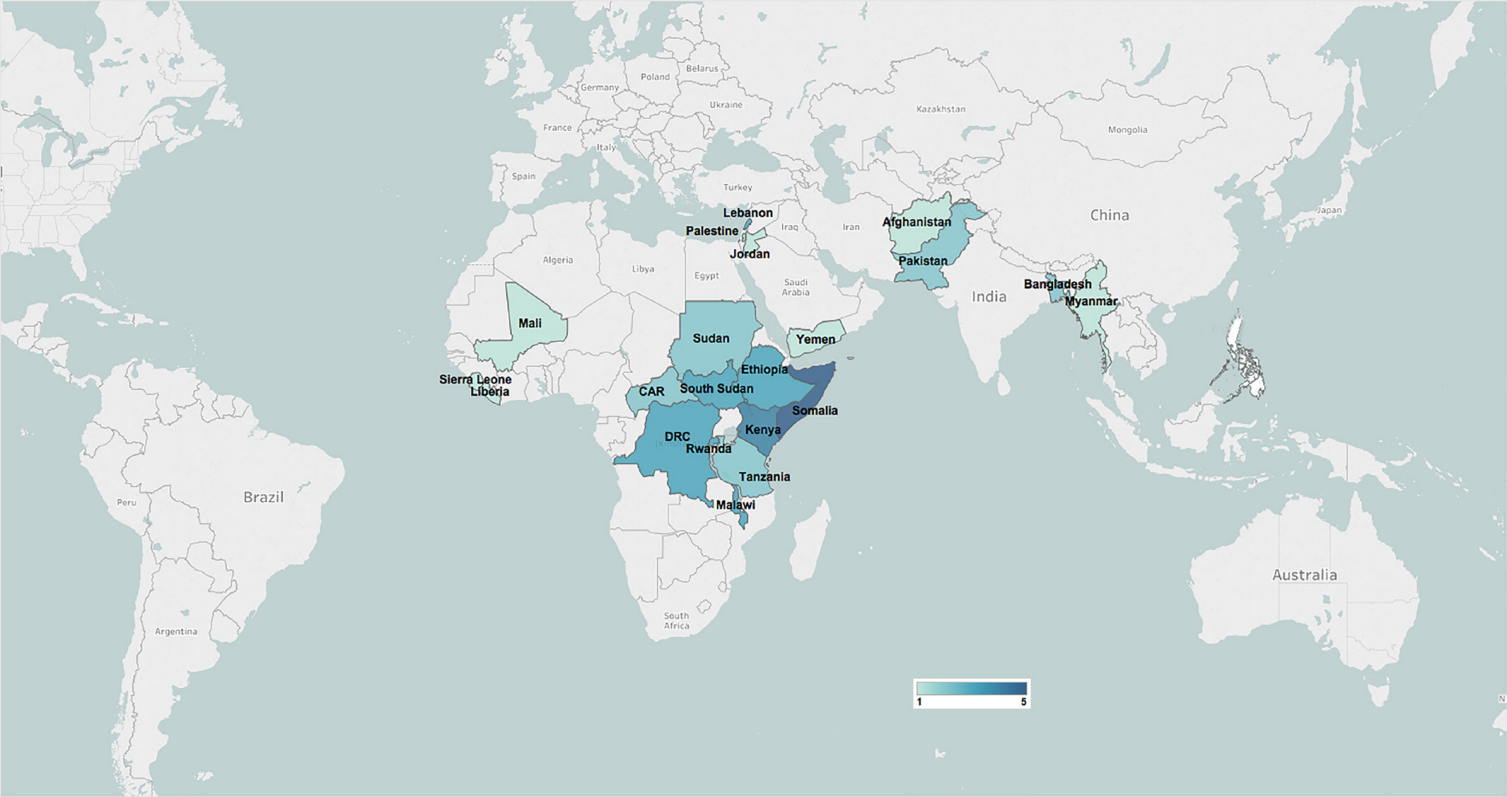
Geographic distribution of the included publications.

Most of the included publications focused on WASH interventions delivered in Sub-Saharan Africa (42/58, 72%), including six focused on interventions targeting women or children specifically (6/42, 14%, figure 3). None of the included publications focused on countries in the Latin America and Caribbean region or in the Europe and Central Asia region. With respect to population displacement status, over 60% of included publications reported on interventions delivered in refugee populations (36/58, 62%), 17% (6/36) of which targeted refugee children, adolescents or pregnant and lactating women (PLW). Almost half of the included publications reported on internally displaced populations (28/58, 48%), with reports of interventions specifically targeting women or child IDPs accounting for a quarter of these (7/28). The delivery of WASH interventions in non-displaced populations was reported in only five publications, of which 60% focused on interventions targeted to women and children (3/5).

**Figure 4:**
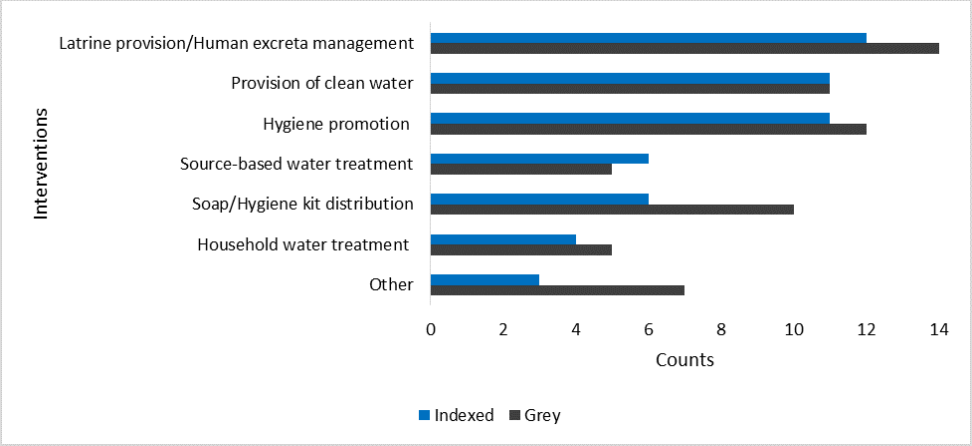
Frequency of interventions reported in the indexed and grey literature.

Water interventions reported in the literature were those aimed at improving the quality or quantity/supply of clean water and included the provision of clean water, household water treatment and source-based water treatment. Sanitation interventions were designed to improve safe excreta disposal and included the provision of latrines or latrine alternatives. Hygiene interventions included the distribution of soap or hygiene kits and hygiene promotion activities. Other interventions included bans on the sale of cooked food and ice blocks,^18^ inspection of township shops and markets to ensure compliance with hygiene practices,^18^ provision of water storage kits to health facilities^19^ and general water and sanitation infrastructure^20^ or services.^21 22^ Within the indexed literature, hygiene promotion interventions were reported in 43% (11/30) of publications, and latrine provision was reported in 40% (12/30) of publications (figure 4). In the grey literature, latrine provision (14/28, 50%) and hygiene promotion (12/28, 43%) were most commonly reported.

Overall, interventions specifically targeted at women or children were captured in 11 (11/58, 19%) publications included in this review.^19 23–32^ Soap/hygiene kit distribution interventions were reported most frequently (6/11, 54%) for these targeted populations, reaching children under 5, adolescents, PLW, and other women.^23 27–30 32^ Two publications reported interventions designed to increase water supply for children,^19 26^ while a single publication reported on this class of intervention for PLW.^19^ Hygiene promotion^24 25 28^ and latrine provision^19 26 31^ were each reported in three publications, aimed at benefiting children and adolescents.

### Delivery characteristics of reported interventions

Here we synthesise retrieved information about the WASH interventions reported to have been delivered to conflict-affected women and children, and the sites and personnel that were used to deliver those interventions.

### Water quality interventions

#### Household water treatment

Nine publications^33–41^ reported on the delivery of water treatment interventions at the household level, including the use of chlorine-based products, storage containers or water filtration systems. Specific interventions included hand pump filters, improved storage containers (jerricans,^38 40^ constricted opening 20 L containers^33^), disinfection of water containers with 5% chlorine solution^34^ and provision of chlorine for disinfection of water.^41^ The provision of chlorine-based water treatment interventions was facilitated by NGO/UN agency staff targeted at the general population living in camp^33–35 38 41^ and non-camp settings.^38 40 41^ Non-chlorine-based water treatment methods were accessible at clinics, home, community spaces and water distribution points. NGO/UN agency staff or researchers were reported to be involved in the delivery of most household water treatment interventions.^33 35 36 38–40^ The delivery of water storage vessels to camp-based refugees in South Sudan,^37^ as well as the distribution of products for point of use water treatment to camp-based refugees in Kenya were facilitated by community health workers (CHWs).^42^ None of the included household water treatment interventions were reported to be targeted at women or children specifically.

#### Source-based water treatment

Eleven publications reported on the delivery of source-based water treatment.^20 21 39 43–51^ Delivery site and personnel were predominantly unreported, but one publication conducted in Pakistan reported the use of tanks to deliver chlorine-treated water to IDPs in community/market settings (online supplementary appendix C).^21^ None of these interventions were reported to be targeted at women or children.

### Water quantity/supply interventions

#### Provision of clean water

Twenty-two publications reported on the provision of safe water for cooking/drinking and general use,^19–21 25 26 36 37 39 41 42 46 47 50–59^ two of which explicitly targeted women and children.^19 26^ We captured 15 studies that reported the use of NGO/UN agency staff to increase access to clean water.^19–21 25 26 36 37 39 41 46 47 50 51 54–57^ Community^21 42 54 56 57^ and water distribution sites (eg, water tanks installed on vans, water supply facilities and water points)^20 25 36 39 46 47 51 55 58 59^ were the most commonly reported intervention delivery sites. In the Democratic Republic of Congo, internally displaced children under 5 and PLW had clean water provided through water sites constructed by NGO/UN agency staff in health centres.^19^

### Sanitation interventions

#### Latrine provision/human excreta management

The provision of latrines was reported in 26 (26/58, 45%) publications within our review,^18 20 21 26 31 36 37 39 41 42 44–46 48 49 51 53–55 60–65^ three of which targeted children and adolescents.^19 26 31^ In Somalia, for example, staff from the Formal Education Network for Private Schools alongside NGO/UN agency staff built sanitation facilities in schools for non-displaced children and adolescents.^26^ Three publications reported on the building of separate sanitation facilities for males and females^21 31 65^ and one of these additionally incorporated hand washing stations into the construction.^21^ An intervention in Pakistan targeted at IDP and refugee children and adolescents used NGO/UN agency staff to build separate facilities for males and females, fitting the inside of the latrine doors with locks for added safety in community/market spaces as well at schools.^31^ In Kenya, refugees living in camps were provided with waste disposal bins designated for feminine hygiene products and non-organic solid waste.^64^ The intervention was delivered by NGO/UN agency staff at the beneficiaries’ homes.^64^ Overall, site of delivery was often unreported, but among those publications that did report delivery sites, these included care centres,^18^ mobile clinics,^53^ road stations,^53^ schools,^26 31^ households^41 45 64^ and community settings.^21 31 37 41 42 48 54 60–63 65^ A single publication reported the use of sanitary workers in latrine construction efforts.^21^ Interventions generally described the construction or restoration of latrines for conflict-affected populations, with minimal details on the types of latrines provided, and delivery personnel was often unreported.

### Hygiene interventions

#### Soap/hygiene kit distribution

Of the 58 publications included, 16 reported on the delivery of soap/hygiene kit distribution interventions, with six reporting on interventions targeted specifically at women or children.^23 27–30 32^ Half of these (3/6) reported on refugees,^23 27 28^ four on IDP^23 29 30 32^ populations, two reported on non-displaced populations^29 30^ and one reported on a host^28^ population. NGO/UN agencies were reported as a delivery platform in all publications, while health system–NGO partnerships were additionally reported in three publications.^23 37 66^ A single publication reported implementation collaboration between NGO/UN agencies, the healthcare system, the education system, a faith-based system and a mass media platform.^28^ Health workers^23^ and NGO/UN agency staff^27–30 32^ were involved in soap/hygiene kit distribution to conflict-afflicted children, adolescents and women. A study conducted in Lebanon utilised county officers alongside NGO/UN agency staff and other partners to deliver dignity kits (including sanitary towels, women's underwear, antibacterial soap, solar flashlight, wet wipes, headband/headscarf, cotton/polyester overcoats, socks, multipurpose cloth, fabric bag and a packing carton) to adolescents 10 years of age and older.^28^ Children, adolescents, adult women and PLW accessed soap and/or hygiene kits at clinics,^23 40^ health posts,^27^ community spaces, schools and hospitals.^28^ The distribution of menstrual hygiene kits to IDPs and refugees was reported in a single publication in Cameroon.^51^ General population interventions in this category included provision of soap,^30 36 37 42 61 67^ establishing hand washing stations with soap,^68^ distribution of e-vouchers for hygiene products^57^ and general hygiene kits.^23 27 30 32 38 39 51 66^

#### Hygiene promotion

We identified 23 publications that described the delivery of hygiene promotion interventions, including 19 in Sub-Saharan Africa,^20 25 36 37 39 40 42 43 45 46 48 49 55 66 68–72^ 5 in the Middle East and North Africa region^20 24 28 47 73^ and 2 in East Asia and the Pacific.^46 55^ Six publications described implementing general hygiene promotion activities without further details,^20 28 36 43 45–48 55 71 73^ a single publication described hygiene promotion activities targeted at preventing acute watery diarrhoea,^66^ two publications reported on hygiene promotion messaging to prevent waterborne diseases (malaria, dengue fever)^39^ and cholera,^49^ seven publications discuss hand washing or hand hygiene education,^21 34 40 42 68 70 72^ five report hygiene education,^24 25 37 45 69^ and a single publication describes the promotion of safe practices for latrine construction and usage for the safety of young girls (eg, locks on doors, avoiding young girls going to the facilities alone), combining women’s rights messages with hygiene promotion activities.^28^ Hygiene promotion interventions reported in most publications were delivered to the general population (21/23 publications, 91%), but those reported in two publications targeted specific age groups in host and refugee populations in Lebanon,^24^ and IDPs in Sudan.^25^ One publication in Lebanon targeted children 0–59 months of age as well as PLW.^24^ In Sudan, school-aged IDPs received sanitation and hygiene education in schools.^25^ The delivery platform reported in all hygiene promotion publications, either independently or in collaboration with other implementing agencies was the NGO/UN agency platform, with the existing healthcare system being involved as the delivery platform in nine (50%) publications. Doctors,^24^ nurses,^24^ social workers^24^ and NGO/UN agency staff^25^ were the personnel reported in the delivery of hygiene promotion activities to women and children.

### Other interventions

Ten publications^18–22 36 55 57 74 75^ reported interventions that did not align specifically with one of the seven WASH intervention categories outlined above. These interventions were delivered in parts of East Asia and the Pacific,^18 36 74^ Sub-Saharan Africa,^19 20 22 36^ South Asia, and the Middle East and North Africa region.^20 36 57 75^ Camp-based and non-camp refugees^20 36 57 74 75^ and IDPs,^18–20 22 36 57 75^ host populations,^20 55 74^ returning refugees^20^ and non-displaced persons^18 57^ were beneficiaries of these other WASH-related interventions. A single publication^19^ described the provision of water storage containers to health facilities and while the publication outlines the target population as children and PLW, the storage containers would have also benefitted others. Three publications reported on the utilisation of cash-based interventions for improving access to WASH services.^36 57 75^ The distribution of e-vouchers was reported in Palestine, reaching IDPs and refugees to improve access to hygiene products.^57^ In Jordan, camp-based and non-camp refugees accessed cash assistance at automated teller machines across all governorates in Jordan.^75^ In Somalia, water vouchers to be exchanged in the local markets were provided to vulnerable households to improve access to clean water.^36^ Two unique interventions were reported from a publication reporting on IDPs and non-displaced populations in Papua New Guinea.^18^ One intervention was a ban on the sale of ice blocks and cooked food in response to an outbreak of shigellosis, implemented by civic leaders, police and town council members, with the inspection of food stalls carried out by environmental health officers.^18^ In addition to the bans, inspections were conducted in shops and markets to ensure proper water, sanitation and hygiene practices were in place.^18^ Overall, the delivery platform reported most frequently was the NGO/UN agency platform,^18–21 36 55 57 74 75^ while delivery through the existing healthcare system was reported in a single publication.^22^

### Intervention coverage and effectiveness

The coverage and effectiveness outcomes of interest for this review were those reported for children, adolescents or women. Data stratified by age and gender were rare within the literature however, and we identified only a single publication from which relevant data could be extracted. A randomised controlled trial (RCT) conducted in Malawi in 1993 found that household water treatment in the form of improved 20 L containers with constricted openings reduced diarrhoea incidence by 31.1% in children under 5, with 84.3 diarrhoea episodes reported per 1000 child-months in households with the improved container compared with 122.4 episodes in households without.^33^ As only a single publication presented quantitative estimates we were unable to perform meta-analyses.

### Barriers to and facilitators of intervention delivery to women and children

Key WASH delivery barriers and facilitators, as reported by publication authors, were available in three publications reporting on interventions targeted at women and children. Reported barriers related to inequity of access and poor communication. One publication reported on the construction of separate male and female sanitation facilities in schools in Somalia, to provide extra security for young girls, but the authors noted that a challenge to reaching the target population with this intervention is that boys are often favoured to attend school over their female siblings in this context.^26^ The two other publications suggested that implementing organisations ineffectively communicated the gender-based violence (GBV) programmes available to intended beneficiaries,^28 29^ limiting their uptake. Reported intervention delivery facilitators were the provision of incentives^28^ and local acceptability.^29^ The provision of dignity kits were noted to incentivise the uptake of GBV services in Lebanon. In Somalia, GBV services were more effectively implemented as a result of community trust in UNICEF’s reputation; their involvement appeared to improve acceptability of the programme and encouraged government participation in implementing GBV services.^29^

## Discussion

### Principal findings

Of the 58 publications included in this review on WASH intervention delivery to conflict-affected women and children, the majority were non-research reports of intervention delivery in Sub-Saharan Africa. Only 11 (19%) publications reported on interventions targeted specifically at children,^19 23–26 30 31^ adolescents,^27–29 32^ women^29 30 32^ and/or pregnant and lactating women,^19 24 27 29 30^ with the rest reporting on the delivery of general population or other broadly focused interventions that included women and children among their beneficiaries. Most women-focused or child-focused interventions included the delivery of soap, hygiene or dignity kits, while others included hygiene promotion messaging, or the provision of clean water or latrines for these populations specifically. The involvement of NGO/UN agency staff was cited in most publications reporting on the delivery of WASH interventions targeted at women or children (9/11, 82%),^19 25–32^ while clinics (fixed^19 23 24^ or mobile^28^) and community spaces^28–31^ were most commonly reported as delivery sites. We were able to extract sub-population morbidity and effectiveness data from a single study only, which showed a decrease in diarrhoea incidence among refugee children under 5 associated with the use of an improved water storage container in Malawi.^33^

### Evidence gaps and implications for future research and practice

The findings of our review reveal a number of important gaps in the current evidence on WASH intervention delivery in conflict settings. First, there is very limited information available on the delivery of household water treatment, source-based water treatment or environmental hygiene interventions. The relatively infrequent reporting of these interventions may reflect the prioritisation of other WASH interventions by humanitarian organisations that are less logistically challenging to implement. Environmental hygiene interventions may be particularly challenging, as they require major infrastructure changes, and sufficient funding, whic are both often difficult to secure in conflict settings. New approaches to facilitate the delivery of source-based and household water treatment as well as environmental hygiene interventions are important areas of further investigation, especially given the burden of related diseases in such settings. Overcrowding and water scarcity in conflict settings present optimal conditions for the spread of waterborne diseases, but only about half of the included publications focused on interventions targeting such diseases. Of these, cholera was the most frequently reported (21/58, 36%), followed by diarrhoea (8/58, 14%), with only one publication reporting on interventions to control typhoid,^58^ and one on interventions for hepatitis E.^76^

Secondly, no information or data on WASH intervention delivery were captured in our review from countries in the Latin America and Caribbean region, and among those included publications from South Asia, East Asia and the Pacific, and the Middle East and North Africa region, only a few countries were represented within each region. These patterns indicate that the available literature is not representative in terms of the conflict-affected populations that it covers, suggesting that the WASH needs of women and children have not or are not being sufficiently considered in the humanitarian response in many conflict settings, or that documentation of such consideration is sorely lacking.

Third, we were able to capture only limited information about where and by whom WASH interventions were being delivered to conflict-affected populations, constraining the value of the current literature for informing future strategies for WASH intervention programming. Based on the available information about delivery personnel, a missed opportunity appears to be the limited use of teachers in the delivery of hygiene promotion and soap/hygiene kit distribution for school-aged children. Instilling proper hygiene practices among children and adolescents and providing them with the tools to carry them out (clean water, soap, dignity kits, menstrual sanitation products, etc) can promote improved health and reduce the burden of waterborne and other infectious diseases. Only one study in our included literature reported the use of teachers to deliver WASH interventions, describing their participation in a mass hand washing campaign initiated to prevent cholera in refugee camps in Tanzania.^70^ Additionally of note is the very limited reported use of doctors, nurses and health workers in the delivery of WASH interventions. Doctors and nurses are reported in a single study providing hygiene education to refugee and host population children under 5 and PLW.^24^ Healthcare workers have access to larger numbers of people accessing health services and may therefore be able to reach more at-risk individuals with WASH services. Only seven of the included studies reported on the use of health workers for the delivery of WASH interventions including health promotion, hygiene kit distribution and source-based water treatment, among others.^18 23 43 48 66 68 71^

Finally, the very limited quantitative data available on WASH intervention coverage and effectiveness with respect to women and children is a very important gap in the literature, as cultural norms can impact how WASH services are accessed by women versus men, and the hygiene needs of adolescent girls and boys also differ. These differences must be taken into consideration when delivering interventions in conflict settings to ensure equity. In addition to addressing the need for more rigorous evaluation of WASH interventions generally, an important step moving forward would be to establish reliable estimates on intervention coverage and effectiveness disaggregated by age and gender to ensure the particular needs of children, adolescents and women in conflict settings are being met appropriately.

### Limitations

Given our inclusive eligibility criteria that aimed to capture as much information as possible about intervention delivery, our review included a wider body of literature on WASH interventions in conflict settings than three previously published reviews.^6 77 78^ Despite the wider range of included literature and a specific focus on intervention delivery, our review also has several limitations. The lack of sufficient information on delivery site and personnel for the interventions captured in our included literature makes it difficult to develop recommendations on strategies and approaches for improving WASH intervention coverage in conflict settings generally, with the scarcity of data on WASH interventions targeted at children and adolescents or women making such recommendations for these vulnerable populations specifically even more difficult. From a methodological perspective, our inability to assess non-English publications and our comprehensive but not exhaustive search of the grey literature means that some relevant publications may have been missed. Moreover, it is likely that some of the health and nutrition programming of humanitarian organisations is undocumented altogether, with the details of intervention delivery available in neither the grey nor the indexed literature. This makes it difficult to ascertain whether gaps in the literature reflect deficiencies in current WASH programming in conflict settings, or simply deficiencies in reporting.

## Conclusion

Conflict-affected populations, and particularly women and children, need safe water, adequate sanitation facilities and sufficient supplies to facilitate good hygiene practices, but information on how best to deliver such intervention in such settings is still very limited. Key delivery challenges for interventions targeted at women and children include inequity of access in some areas due to gender norms favouring male access to schooling and thus to school-based interventions, and ineffective communication between implementers and beneficiaries. These challenges and their potential solutions undoubtedly vary by geography and population, but the availability of data and information on WASH intervention delivery and effectiveness from several regions is limited, as are data and information on delivery and effectiveness among women and children specifically. Better documentation of current practice in the field and further research into the relative effectiveness of different delivery strategies are both needed to help overcome existing challenges and improve future WASH programming for women and children in conflict settings.
