# Supplementary material for: Delivering water, sanitation and hygiene interventions to women and children in conflict settings: a systematic review
Source: BMJ Glob Health. 2020 Jul 8;5(Suppl 1):e002064. doi: 10.1136/bmjgh-2019-002064 (PMC7348465; doi:10.1136/bmjgh-2019-002064)
Supplement: Supplementary data [file bmjgh-2019-002064supp001.pdf]

Appendix A. Search strategy for MEDLINE database

I. Conflict related terms

1. Medline

1. disasters/ or emergencies/ or mass casualty incidents/
2. disaster victims/
3. ((disaster or disasters or catastrophe or catastrophes) adj5 (environ* or human or manmade or "man made" or nature or natural or weather)).tw,kf.
4. ("mass casualty" or "mass casualties" or "mass fatalities" or "mass fatality").tw,kf.
5. ((crisis or crises) adj5 (environ* or human or manmade or "man made" or nature or natural or weather)).tw,kf.
6. "warfare and armed conflicts"/ or armed conflicts/ or warfare/ or biological warfare/ or bioterrorism/ or chemical warfare/ or chemical terrorism/ or nuclear warfare/ or psychological warfare/ or war crimes/ or ethnic cleansing/ or genocide/ or holocaust/ or war exposure/ or war-related injuries/
7. afghan campaign 2001-/ or gulf war/ or iraq war, 2003-2011/
8. ("afghan campaign" or "armed conflict" or "armed conflicts" or "gulf war" or "iraq war" or "war time" or "wartime").tw,kf.
9. ((armed or zone or political or civil) adj3 (conflict or conflicts or attack or attacks or war or wars or "no fly")).tw,kf.
10. ("war related injuries" or "war related traumas" or "war related injury" or "war related trauma").tw,kf.
11. ("militant group" or "militant groups" or "militant organization" or "militant organizations" or "militant organisation" or "militant organisations").tw,kf.
12. ("biological terrorism" or bioterrorism or biowarfare or "chemical terrorism" or "ethnic cleansing" or "ethnic cleansings" or "gas poisoning" or genocide or holocaust or holocausts or "nuclear terrorism" or "war exposure" or "war exposures").tw,kf.
13. Disaster Medicine/
14. disease outbreaks/
15. Emergency Medical Services/
16. ((emergency or emergencies) adj5 (environ* or human or manmade or "man made" or nature or natural or weather)).tw,kf.
17. Starvation/
18. (famine or famines or starvation or starvations).tw,kf.
19. cyclonic storms/ or droughts/ or floods/ or tornadoes/ or tidal waves/
20. avalanches/ or earthquakes/ or landslides/ or tidal waves/ or tsunamis/ or volcanic eruptions/
21. (avalanche or avalanches or cyclone or cyclones or drought or droughts or earthquake or earthquakes or flood or flooded or flooding or floods or hurricane or hurricanes or landslide or landslides or "land slide" or "land slides" or mudslide or mudslides or "mud slide" or "mud slides" or storm or storms or tornado or tornadoes or tsunami or tsunamis or typhoon or typhoons or "volcanic ash" or "volcanic eruption" or "volcanic eruptions" or "volcanic gases").tw,kf.
22. refugees/
23. (evacuee or evacuees or refugee or refugees or squatter or squatters or transients).tw,kf.
24. relief work/ or rescue work/
25. ((rescue or relief or aid) adj (plan or plans or activity or activities or agency or agencies)).tw,kf.
26. ("aid plan" or "aid work" or "relief plan" or "relief work" or "rescue plan" or "rescue work").tw,kf.
27. ((staff or staffs or worker or workers) adj3 (relief or aid)).tw,kf.
28. (humanitarian assistance or humanitarian assistances or relief work or relief works).tw,kf.

29. (humanitarian adj2 (aid or response or relief or crisis or crises or emergency or emergencies or disaster or disasters)).tw,kf.
30. Altruism/
31. (humanitarianism or altruism).tw,kf.
32. ("displaced children" or "displaced families" or "displaced family" or "displaced individuals" or "displaced internally" or "displaced men" or "displaced people" or "displaced peoples" or "displaced person" or "displaced persons" or "displaced population" or "displaced populations" or "displaced women" or "forced displacement" or "forced displacements" or "internal displaced" or "internal displacement" or "internally displaced" or "population displaced" or "population displacement").tw,kf.
33. (((camp or camps) and displac*) or "protected village*").tw,kf.
34. (victim or victims).tw,kf.
35. rubble.tw,kf.
36. or/1-35

II. Population of interest

1. MEDLINE

37. adolescent/ or young adult/

38. (adolescence or adolescent or adolescents or teen* or youth or youths or "young adult" or "young adults").tw,kf.

39. Pregnant Women/

40. exp pregnancy/

41. (expectant or expectancy or gravid* or pregnant or pregnancies or pregnancy).tw,kf.

42. ("mother to be" or "mothers to be").tw,kf.

43. (prenatal or "pre natal").mp.

44. (perinatal or "peri natal").mp.

45. ((trimester or trimesters) adj3 (first or second or mid or third or final or "1st" or "2nd" or "3rd")).tw,kf.

46. (midtrimester or midtrimesters or "early placental phase" or "early placental phases").tw,kf.

47. exp Delivery, Obstetric/

48. ((labor or labour) adj5 (birth* or breech or childbirth or childbirths or complicat* or difficult or early or easy or induce* or induction or late or obstetric* or onset or pregnan* or present*)).tw,kf.

49. parturients.tw,kf.

50. (birth or births or childbirth or childbirths or parturition or parturitions).tw,kf.

51. ("abdominal deliveries" or "abdominal delivery" or "c-section" or "c-sections" or caesarean or caesareans or cesarean or cesareans or "postcesarean section" or "postcaesarean section").tw,kf.

52. exp Abortion, Induced/

53. (abortion or abortions or embryotomies or embryotomy or "postconception fertility control").tw,kf.

54. ((pregnancy or pregnancies) adj3 terminat*).tw,kf.

55. "sexually active".tw,kf.

56. child/ or child, preschool/ or infant/ or infant, newborn/ or infant, low birth weight/ or infant, small for gestational age/ or infant, very low birth weight/ or infant, extremely low birth weight/ or infant, postmature/ or infant, premature/ or infant, extremely premature/

57. (infan* or newborn* or "new born*" or neonat* or baby* or babies or toddler* or boy or boys or boyfriend or boyhood or girl* or kid or kids or child* or pediatric* or paediatric* or peadiatric* or prematur* or preterm*).mp. or school*.tw.

58. refugees/

59. (refugee or refugees).tw,kf.

60. or/37-59

61. 36 and 60

III. Domain specific terms – WASH

1. MEDLINE

62. Hygiene/ or Hand Hygiene/ or Disinfection/ or Hand Disinfection/ or Sanitation/ or Sanitary Engineering/ or Toilet Facilities/ or Waste Disposal, Fluid/

63. (hygiene or hygienic or "hand sanitisation" or "hand sanitization" or "hand washing" or handwashing or disinfect or disinfected or disinfection or detergent or soap or soaps or sanitary or sanitation or "public facilities" or latrine or latrines or "septic tank" or "septic tanks" or toilet or toilets or "non food item" or "non food items" or "core relief item" or "core relief items").tw,kf.

64. Feces/

65. (faeces or feces or "faecal excretion*" or "fecal excretion*" or defecat* or defaecat* or stool or stools or "faecal matter" or "faecal matters" or "fecal matter" or "fecal matters" or "fecal oral" or "faecal oral").tw,kf.

66. Waste Water/ or Sewage/

67. ("waste water" or "waste waters" or wastewater or wastewaters or sewage or sludge or sewerage or "human excreta disposal" or "excreta management").tw,kf.

68. Refuse Disposal/

69. ("garbage collection*" or "garbage disposal*" or "refuse collection*" or "refuse disposal*" or "trash collection*" or "waste disposal*" or "waste treatment*").tw,kf.

70. Drinking Water/

71. ("clean water" or drinkwater or "drink* water" or "piped water" or "potable water" or "fresh water" or "private water" or "public water").tw,kf.

72. Water Supply/

73. ("water supply" or "water supplies" or "water source" or "water sources" or "water provision*" or "rain water" or rainwater).tw,kf.

74. Ground Water/

75. (aquifer or aquifers or "bore well*" or borewell* or "ground water*" or groundwater* or "water table" or "well water*" or wellwater*).tw,kf.

76. Water Pollution/ or Water Quality/

77. ("water quality" or "water pollut*").tw,kf.

78. watsan.tw,kf.

79. "point of use".tw,kf.

80. or/62-79

81. 61 and 80

82. limit 81 to dc=20170530-20180331

83. ("2017 05 30*" or "2017 05 31*" or "2017 06*" or "2017 07*" or "2017 08*" or "2017 09*" or "2017 10*" or "2017 11*" or "2017 12*" or "2018 01*" or "2018 02*" or "2018 03*").dt.

84. 81 and 83

85. 82 or 84

~~~ End of Appendix ~~~
~~~

Appendix B. Characteristics of indexed publications

Author, Pub Year	Report Type	Country	Target Population	Displacement status	Setting	Interventions	Intervention start-year	Delivery Platform	Delivery Approach	Delivery Site	Delivery Personnel
Ahoua, 2006	Obs. study	Congo (Democratic Republic of)	All ages	IDPs	Camp	Latrine provision, Provision of clean water	2005	NGO/UN agencies	NR	Community/ markets	NGO/ UN Agency Staff
Altmann, 2017	Non-research	Yemen	All ages	NR	NR	Hygiene promotion, Provision of clean water, Source-based water treatment	2016	NGO/UN agencies, Healthcare system	Centre-based, Outreach	Water distribution point, Home	CHWs, NGO/ UN Agency Staff
WHO, 1994	Non-research	Rwanda, Tanzania, Congo(Democratic Republic of)	All ages	IDPs, Refugees	Camp	Provision of clean water	1994	NGO/UN agencies	NR	NR	NR
Benjamin, 1996	Non-research	Tanzania	All ages	Refugees	Camp	Latrine provision	1994	NGO/UN agencies	Outreach	Community/ markets	NR

Benny, 2014	Obs. study	Papua New Guinea	All ages	IDPs, Not displaced	Camp	Ban on the sale of cooked food and ice blocks, Hygiene practice compliance inspections (township shops and markets), Latrine provision	2013	Healthcare system	Centre-based	Clinics, Community/ markets	Health workers, Civic leaders
Bile, 2011	Non-research	Pakistan	All ages	IDPs	Camp, Dispersed	Hygiene promotion, Latrine provision, Provision of clean water, Source-based water treatment	NR	NGO/UN agencies, Healthcare system	Centre-based, Outreach	First level care facilities, Community/ markets, Camps - not otherwise specified	CHWs, NGO /UN Agency staff
Brown, 1997	Non-research	Rwanda	All ages	Returning refugees	Rural	Latrine provision, Provision of clean water	1996	NGO/UN agencies, Healthcare system	Centre-based, Outreach	Clinic, Mobile clinics, Health posts	NR
Chan, 2018	Non-research	Bangladesh	All ages	Refugees	Camp, Dispersed	Provision of clean water	2017	NGO/UN agencies	Centre-based	Water distribution point	NR
Doocy, 2006	RCT	Liberia	All ages	IDPs	Camp	Household water treatment	2004	NGO/UN agencies	NR	Home	NGO/ UN Agency staff

Eltom, 2001	Non-research	Afghanistan	All ages	IDPs, Returning refugees	Camp	Latrine provision, Soap/hygiene kit distribution	2001	NGO/UN agencies	NR	Community/ markets	NGO/ UN Agency staff, CHWs
Guerin, 2004	Obs. study	Sierra Leone	All ages	NR	NR	Hygiene promotion	1999	NGO/UN agencies	Centre-based, Outreach	Clinics, Community/ markets	Health workers
Hatch, 1994	Obs. study	Malawi	All ages	Refugees	Camp, Dispersed	Hygiene promotion, Source-based water treatment	1988	NGO/UN agencies, Healthcare system	Outreach	Hospital	Health workers
Husain, 2015	Mixed methods	Ethiopia	All ages	Refugees	Camp	Hygiene promotion, Soap/hygiene kit distribution	2012	NGO/UN agencies	Outreach	Home	Health workers
Iijima, 1994	Obs. study	Kenya	All ages	Hosts, Refugees	NR	Hygiene promotion	1994	NGO/UN agencies	NR	NR	NR
Kajeechiwa, 2016	Ob. study	Thailand	All ages	Refugees	Dispersed	Provision of clean water	2013	NGO/UN agencies, Research	Centre-based	Community/ market	NGO/ UN Agency staff, Researchers
Kur, 2009	Obs. study	South Sudan	All ages	IDPs, Refugees	Camp, Dispersed	Hygiene promotion, Latrine provision, Provision of clean water, Source-based	2007	NGO/UN agencies, Healthcare system	Centre-based, Outreach	Community/ market, Home, Camps - not otherwise specified, Water distribution point	NGO/ UN Agency Staff, Health workers

						water treatment					
Mahamud, 2012	Obs. study	Kenya	All ages	Refugees	Camp	Household water treatment, Hygiene promotion, Latrine provision, Provision of clean water Screening for referral, Soap/hygiene kit distribution	2009	NGO/UN agencies	Outreach	Community/ markets	CHWs
Matthys, 1998	Non-research	Congo (Democratic Republic of)	All ages	Refugees	Camp	Latrine provision, Source-based water treatment	1997	NGO/UN agencies	Centre-based	NR	NR
Milton, 2017	Non-research	Bangladesh	All ages	Refugees	Camp	Provision of clean water	NR	NGO/UN agencies, Healthcare system	Centre-based	Water distribution point	NR
Nahimana, 2017	Obs. study	Rwanda	All ages	Refugees	Camp	Hygiene promotion, Latrine provision, Source-based water treatment	2016	NGO/UN agencies, Healthcare system	Centre-based, Outreach	Community /markets, Home	Health workers, CHWs

Nyoka, 2017	Mixed methods	Kenya	All ages	Refugees	Camp	Latrine provision/ human excreta management	2014	NGO/UN agencies	Outreach	Home	NGO/ UN Agency staff
Obol, 2013	Obs. study	Uganda	All ages	IDPs	Camp	Water and sanitation services	2009	NGO/UN agencies	Outreach	NR	NR
Peterson, 1998	Obs. study	Malawi	All ages	Refugees	Camp	Soap/hygiene kit distribution	1993	NGO/UN agencies	Outreach	NR	NR
Plummer, 1995	Non-research	Tanzania	All ages	Refugees	Camp	Hygiene promotion	1994	NGO/UN agencies	Outreach	NR	CHWs, Religious Leaders, Teachers
Roberts, 2001	RCT	Malawi	All ages	Refugees	Camp	Household water treatment	1993	NGO/UN agencies	Outreach	Home	NGO/ UN Agency staff
Rull, 2018	Non-research	South Sudan	All ages	IDPs	Camp	Provision of clean water, Source-based water treatment	2013	NGO/UN agencies	Centre-based	NR	NGO/ UN Agency staff
Schmitt, 2017	Qualitative study	Burma (Myanmar)	Adoles cents girls (10-19 years old), Women (20-49 years old)	IDPs	Camp	Soap/hygiene kit distribution	2015	NGO/UN agencies	NR	NR	NGO/ UN Agency staff

Shultz, 2009	Obs. study	Kenya	All ages	Refugees	Camp	Hygiene promotion	2005	NGO/UN agencies	NR	NR	NR
Thomson, 2013	Obs. study	South Sudan	All ages	Refugees	Camp	Household water treatment, Hygiene Promotion, Latrine Provision, Provision of clean water, Screening for referral, Soap/hygiene kit distribution	2012	NGO/UN agencies, Healthcare System	Centre-based, Outreach	Clinics, Home, Community/ market	CHWs
Walden, 2005	Obs. study	Sudan	All ages	IDPs	Camp	Household water treatment, Hygiene promotion	2004	NGO/UN Agencies	Outreach	Water distribution point, Home	NGO /UN Agency staff

Appendix D. Characteristics of grey literature publications

Author, Pub Year	Report Type	Country	Target Populatio	Displacement status	Setting	Interventions	Intervention start-year	Delivery Platform	Delivery Approach	Delivery Site	Delivery Personnel
IRC, 2016	Non-research	Ethiopia	All ages	Refugees	Camp	Hygiene promotion, Latrine provision	NR	NGO/UN agencies	Outreach	Community/ market	NGO/UN Agency staff
UNHCR, 2018	Non-research	Rwanda	All ages	Refugees	Camp	Latrine provision	2016	NGO/UN agencies	Centre-based	Community/ market	NGO/UN Agency staff
Giordano, 2017	Non-research	Jordan	All ages	Refugees	Camp, Dispersed	Cash based intervention/ assistance	2016	NGO/UN agencies	Centre-based	Community/ markets	NGO/UN Agency staff
Richardson, 2013	Non-research	Ethiopia	All ages	Refugees	Camp	Provision of clean water, Latrine provision	2011	NGO/UN agencies	NR	Community/ markets	NGO/UN Agency staff

Coinco, 2014	Non-research	Sudan	13mo-19yrs, all ages	IDPs	NR	Hygiene promotion, Provision of clean water	2015	NGO/UN agencies	Centre-based	Schools, Water distribution point	NGO/UN Agency staff, Community members
Waddah, 2016	Non-research	Palestine	All ages	IDPs, Refugees	Dispersed	Provision of clean water, e-voucher for hygiene products and WASH support	2014	NGO/UN agencies	Centre-based, Outreach	Home, Community/ markets	NGO/UN Agency staff
Leonardi, 2013	Non-research	Mali	13mo-14yrs	IDPs, Refugees	Dispersed	Soap/hygiene kit distribution	2012	Healthcare system, NGO/UN agencies	Centre-based	Clinics	Health workers
UNICEF,2016 (A)	Non-research	Pakistan	13mo-19yrs	IDPs, Refugees	Camp	Latrine provision	2011	Education, NGO/UN agencies	Centre-based, Outreach	Community/ markets, Schools	NGO/UN Agency staff

UNICEF, 2016 (B)	Non-research	Lebanon	Adolescents ≥10yrs	Host, Refugees	NR	Hygiene promotion, Soap/hygiene kit distribution	2015	Education, Faith-based system, Healthcare system, Mass media, NGO/UN agencies	Centre-based, Outreach	Camps, Electronic/ print, Health posts, Hospitals, Mobile clinics, Place of worship, Schools, Community/ markets, Home	NGO/UN Agency staff, Lebanon Country Officers, Implementing partners
Apiyo, 2014	Non-research	Somalia	All ages	Host, IDPs, Returning Refugees	Camp, Dispersed	Household water treatment, Soap/hygiene kit distribution	2011	NGO/UN agencies	Centre-based	Water distribution point	NGO/UN Agency staff
UNICEF, 2016 (C)	Non-research	Somalia	Adolescents 10-19yrs, Women	IDPs, Not displaced	Camp, Dispersed	Soap/hygiene kit distribution	2011	NGO/UN agencies	Centre-based	Community/ markets	NGO/UN Agency staff

			≥20yrs, PLW								
UNICEF, 2016 (D)	Non-research	Central African Republic	Children 13mo-19yrs, Women ≥20yrs, PLW	IDPs, Not displaced	Camp, Dispersed	Soap/hygiene kit distribution	2014	NGO/UN agencies	Centre-based	Community/ markets	NGO/UN Agency staff
UNICEF, 2016 (E)	Non-research	Central African Republic	All ages	IDPs	Dispersed	Household water treatment, Hygiene promotion	2013	NGO/UN agencies	Outreach	Camps	NGO/UN Agency staff

Crook, 2018	Non-research	Somalia	All ages	Host, IDPs	Camp, Dispersed	Household water treatment, Provision of clean water, Latrine provision	2016	NGO/UN agencies	Outreach	Community/ markets, Home	NGO/UN Agency staff
Berbari, 2014	Non-research	Lebanon	Children 0-59mo, PLW	Host, Refugees	Camp, Dispersed	Hygiene promotion	2012	Healthcare system, NGO/UN agencies	Centre-based	Clinics	Doctors, Nurses, Social workers
Seguin, 2014	Non-research	Lebanon	Adolescents ≥15yrs, PLW	Refugees	Camp	Soap/hygiene kit distribution	2014	NGO/UN agencies	Outreach	Health posts, ACF tents(Safe Havens)	NGO/UN Agency staff
Tchamba, 2017	Non-research	Congo (Democratic Republic of)	Children 1-59mo, PLW	IDPs	NR	Latrine provision, Provision of clean water, Provision of water storage kits to health centres	2013	Healthcare system, NGO/UN agencies	Centre-based	Clinics	NGO/UN Agency staff

Desie, 2017	Non-research	Somalia	All ages	IDPs, Not displaced	Camp, Dispersed	Hygiene promotion, Soap/hygiene kit distribution	2017	Healthcare system, NGO/UN agencies	Outreach	Mobile clinics	Health workers, Paramedics
Farah, 2014	Non-research	Somalia	Children 5-19yrs	Not displaced	Dispersed	Latrine provision, Provision of clean water	2013	Education, NGO/UN agencies, Research	Centre-based	Schools	NGO/UN Agency staff, Researchers, Formal Education Network for Private Schools staff
Solidarités International, 2018 (A)	Non-research	Lebanon	All ages	Refugees	NR	Hygiene promotion	2016	NGO/UN agencies	Outreach	Community/ market, home	NGO/UN Agency staff
Solidarités International, 2013	Non-research	Thailand, Myanmar, Afghanistan, Lebanon, South Sudan, Central African Republic, Congo (Democratic	All ages	IDPs, refugees	Camps, dispersed	Latrine provision, Household water treatment, Soap/hygiene kit distribution, Provision of clean water, Water vouchers(other), Hygiene promotion	NR	NGO/UN agencies	Mixed	Health centres, Schools, Community/ market, home, water points	NGO/UN Agency staff, refugees and local Thai people

		Republic of), Somalia, Mali, Mauritania									
Solidarités International, 2014	Non-research	Mali, Mauritania, Central African Republic, Cameroon, Chad, South Sudan, Congo (Democratic Republic of), Somalia, Lebanon, Burma (Myanmar), Thailand	All ages	IDPs, host, refugees, returning refugees	Camps, dispersed	Provision of clean water, Latrine provision, Hygiene promotion, Source-based water treatment, Soap/hygiene kit distribution, Household water treatment	NR	NGO/UN agencies	Mixed	Health facilities, Schools, Community/ markets, Mobile cinemas, water outlets at entry/exit of POC sites, home, water points	NGO/UN Agency staff, waste management committee
Solidarités International, 2015	Non-research	Lebanon, Cameroon, Niger, Congo (Democratic Republic of), South Sudan, Somalia, Burma (Myanmar), Thailand, Afghanistan	All ages	IDPs, host, refugees, returning refugees	Camps, dispersed	Provision of clean water, Latrine provision, Hygiene promotion, Disinfected household toilets (other)	NR	NGO/UN agencies	Mixed	Hospitals, Schools, Community/ markets, home, Water points,	NGO/UN Agency staff

Solidarités International, 2016	Non-research	Nigeria, Syria, Mali, Central African Republic, Cameroon, Congo (Democratic Republic of), South Sudan, Bangladesh, Burma (Myanmar), Thailand	All ages	IDPs, host, refugees, returnees	Camps, dispersed	Latrine provision, Provision of clean water, Source-based water treatment, Hygiene promotion	NR	NGO/UN agencies	Mixed	Hospitals, Health centres, Clinics, Schools, Community/ market, Water treatment plant, Water points	NGO/UN Agency staff
Solidarités International, 2017	Non-research	Syria, Iraq, Mali, Central African Republic, Cameroon, South Sudan, Congo (Democratic Republic of), Bangladesh, Burma (Myanmar), Afghanistan	All ages	IDPs, host, refugees, returnees	Camps, dispersed	Source-based water treatment, Hygiene promotion, Water and sanitation infrastructures (other), Provision of clean water, Latrine provision	NR	NGO/UN agencies	Mixed	Water treatment plant, health centres, home, water points, community/ markets, schools	NGO/UN Agency staff

Solidarités International, 2018 (B)	Non-research	Burkina Faso, Syria, Iraq, Yemen, Mali, Central African Republic, Cameroon, Nigeria, South Sudan, Congo (Democratic Republic of), Bangladesh, Burma (Myanmar), Afghanistan	All ages	IDPs, host, refugees, returnees	Camps, dispersed	Provision of clean water, Source-based water treatment, Latrine provision, Soap/hygiene kit distribution	NR	NGO/UN agencies	Mixed	Water points, water treatment plant, Health centres, Community/ markets, home, schools	NGO/UN Agency staff
Cardon, 2018	Non-research	Congo (Democratic Republic of)	All ages	NR	NR	Hygiene promotion, Latrine provision, Source-based water treatment	2017	NGO/UN agencies	Mixed	Home, Community /markets, Health centres, Schools, Water points	NGO/UN Agency staff, Community relays
Cavalazzi, 2016	Non-research	Thailand	All ages	Host, refugees	Camps	Sludge treatment unit construction(other)	NR	NGO/UN agencies	Outreach	Sludge treatment sites	NGO/UN Agency staff
